# Editorial: Rethinking addiction: technological advances, mechanistic insights, and the re-evaluation of substance use disorders

**DOI:** 10.3389/fnbeh.2026.2009605

**Published:** 2026-10-19

**Authors:** Emmanuel Darcq, W. Matt Howe, Celine Nicolas, Victor Philippe Mathis

**Affiliations:** 1 INSERM UMR-S1329, Strasbourg Translational Neuroscience & Psychiatry, University of Strasbourg, Strasbourg, France; 2 Fralin Biomedical Research Institute, Virginia Tech, Roanoke, VA, United States; 3 School of Neuroscience, Virginia Tech, Blacksburg, VA, United States; 4 University of Bordeaux, INSERM 1215, Neurocentre Magendie, Bordeaux, France

**Keywords:** addiction, central mechanisms, diagnostic, peripheral biomarkers, substance use disorders

Addiction research has entered an era of rapid conceptual advances seeded by recent methodological innovations. These advances have guided the field of addiction neuroscience from broad descriptions of altered reward processing toward increasingly granular accounts of the circuits, cellular populations, molecular mechanisms, and behavioral processes that underwrite substance use disorders (SUDs) (Venniro et al., 2020; Mathis et al., 2025; Nestler, 2025). At the same time, the concept of addiction itself has expanded from an exclusive focus on impact of psychotropic drugs to encapsulating problematic behavioral patterns like gambling, sex, screen time, and food intake. This expansion raises many important questions, including: What exactly defines addiction? Do all forms of addiction stem of pathological patterns of activity within overlapping, or distinct, brain circuit mechanisms? This Research Topic was conceived at the intersection of these developments. Together, these contributions show how technological innovation is uncovering new mechanisms of addiction and improve diagnostic and definition of addictive disorders.

## From better tools to better concepts

One of the most important developments in addiction neuroscience has been the ability to measure behavior and neural activity with unprecedented temporal, chemical, and spatial resolution. Optical tools including genetically encoded biosensors, automated behavioral tracking, and computational analyses have allowed researchers to move beyond relatively coarse behavioral endpoints and examine the dynamic relationship between brain activity and behavior (Conlisk et al., 2023; Dong et al., 2024; Lüscher et al., 2026).

This is particularly important because addiction is not a static state, but a Research Topic of dynamic processes including drug use, seeking, withdrawal, craving, and relapse that are shaped by internal as well as external cues and readout in an environment enriched with competing rewards. Continuous monitoring and increasingly sophisticated behavioral models therefore offer opportunities to capture processes that conventional experimental designs may overlook. In that regard, Mathis et al. introduce a novel, automated, and unbiased approach to characterize the emergence of social deficits during morphine abstinence in mice. By providing long-term longitudinal and quantitative assessment of social behaviors, this work illustrates how improved behavioral phenotyping can reveal dimensions of withdrawal that may remain invisible to conventional assays. In humans, Yilmazer et al. investigate the potential use of new neurophysiological biomarkers to track gambling disorder, specifically theta cordance. Their approach exemplifies how analytical methods like Granger Causality can be leveraged to potentially improve diagnosis, and guide treatment implementation.

Importantly, methodological innovation and basic scientific discovery are not sufficient to revolutionize clinical implementations. Just as increasingly detailed behavioral phenotyping does not necessarily translate into a mechanistic understanding of addiction, identifying specific patterns of neural circuit activity that correlate with addictive states does not reveal the core cognitive disruptions that seed maladaptive behaviors. The challenge is to determine which measurements meaningfully explain the emergence and persistence of maladaptive behaviors and translate this understanding to new diagnostic and prognostic tools.

In this spirit, Insalaco et al. examined longitudinal changes in physiological and mental-health functioning among individuals receiving treatment for SUDs. Their study highlights the importance of following changes in addiction-related outcomes over time rather, thereby providing insights into the dynamics of SUD as a clinical condition that can be used to tailor therapeutic interventions at different points in its trajectory. Similarly, Allache et al. propose craving as a potential transdiagnostic marker for both substance and behavioral addictions. Their perspective raises an important possibility: rather than defining addiction according to the substance or behavior involved, reorienting to core mechanisms shared across different forms of pathological behavior. Beyond cognitive lynchpins, Tyrode et al., suggest that gut microbiota signatures could be predictive of both cocaine use and binge eating disorders. This viewpoint extends addiction research beyond the brain by emphasizing the potential contribution of peripheral biological systems, and reinforce that distinct disorders may share biological signatures. Together, a re-evaluation of the concept of addiction might open new vision and strategies to study and treat addiction. With this in mind, the review by O'Connor revisits the controversial concept of food addiction in the context of GLP-1-based obesity pharmacotherapy. By examining the overlap between neural pathways regulating feeding and those implicated in substance use, it illustrates both the value and the limitations of identifying shared reward mechanisms across disorders but also demonstrate how societal motives must be considered in the evolution of the concept of addiction (O'Connor).

Together, the contributions featured in this Research Topic wed findings from both animal and human research, experimental as well as clinical investigations, and multiple subtypes of addiction-like behavioral disorders. Despite their diversity, they converge on a common message: understanding addiction requires studying dynamic interactions between biological mechanisms, behavior, and context.

## Rethinking addiction

As highlighted by the studies included in this Research Topic, new vistas in addiction research require the field move beyond emphasizing effects on singular behavioral and neural endpoints. Food and drugs, for example, engage overlapping motivational systems, but feeding is also embedded within metabolic regulation and physiological homeostasis. Therefore, problematic eating behaviors necessarily manifest in a manner that will have overlapping, and unique, behavioral and mechanistic underpinnings relative to psychoactive drugs. It's important to acknowledge these domain specific nuances, as well as that not every reward-based behavior necessarily leads to addiction. Indeed, true progress will likely require a greater, field-wide acknowledgment of the complex interplay of environmental, cognitive, neurochemical, and genetic predispositions that seed addiction in certain individuals.

The contributions to this Research Topic highlight three complementary shifts that are increasingly shaping addiction research: from static measures to dynamic trajectories, from isolated brain circuits to interacting biological systems, and from substance-centered definitions to mechanistically informed models of maladaptive behavior.

The future of addiction research will not be defined solely by our ability to measure more molecules, record more neurons, or quantify more behavioral dimensions. Its progress will depend on our ability to use these technologies to ask better questions and improve our analysis and interpretation.

Rethinking addiction therefore does not necessarily mean expanding its definition. It may instead mean refining it: identifying when, why, and for whom a behavior becomes persistent, maladaptive, and difficult to control. This conceptual refinement, supported by increasingly sophisticated technologies and integrative approaches, may ultimately provide a stronger foundation for more precise diagnosis, prevention, and treatment.
